# Commentary: Exploratory data analysis

**DOI:** 10.3389/fpsyg.2015.01247

**Published:** 2015-08-20

**Authors:** Brian D. Haig

**Affiliations:** Department of Psychology, University of CanterburyChristchurch, New Zealand

**Keywords:** exploratory data analysis, abduction, phenomena detection

**A commentary on**

**“Exploratory data analysis,” in *Handbook of Psychology, 2nd Edn***.

*by Behrens, J. T., Dicerbo, K. E., Yel, N., and Levy, R. (2013). Vol. 2, eds J. A. Schinka, W. F. Velicer, and I. B. Weiner (Hoboken, NJ: Wiley), 34–70*.

Despite the importance of exploratory data analysis (EDA) in statistics and science, few people have worked on its philosophical foundations. In psychology, the present author (Haig, 2012; Behrens et al., 2013) have commented on philosophical aspects of EDA. They hold contrasting views about the appropriateness of abductive reasoning as a core component of the philosophy of EDA. Behrens and his co-authors think that abduction provides the “core logic” of EDA. I disagree. In this commentary, I say why I think their position is mistaken, and that their charge that mine is “a particularly disturbing” view of EDA is unfounded.

Abduction as a form of inference is not well-known in academic circles. Broadly speaking, abduction is concerned with the generation and evaluation of *explanatory* hypotheses. In this sense, it contrasts with the more familiar ideas of inductive and deductive inference. Behrens et al. begin by taking their cue from Charles Peirce, and state that abduction is the form of inference involved in generating new ideas or hypotheses. However, surprisingly, Behrens et al. then elect to follow Josephson and Josephson (1994), and characterize abductive inference with the following pattern of reasoning (p. 39):

D is a collection of data (facts, observations, givens).

Hypothesis H explains D (would if true, explain D).

No other hypothesis explains D as well as H does.

Therefore, H is probably correct.

Patently, this argument schema does not describe the abductive process of *hypothesis generation*. Instead, it characterizes the abductive form of reasoning known as *inference to the best explanation*. Inference to the best explanation is used in science to appraise competing theories in terms of their explanatory goodness (Thagard, 1992). In order for the schema to capture abductive hypothesis generation, the third premise, which refers to competing hypotheses, would have to be deleted, and the conclusion would be amended to say that the hypothesis in question was initially plausible, not probably correct.

It is important to differentiate between the abductive generation of hypotheses, and their comparative appraisal in terms of inference to the best explanation. They are discernably different phases of theory construction. By choosing inference to the best explanation, Behrens et al. adopt a conception of abduction that is ill-suited to explicating the process of idea generation, whether it is pattern identification through EDA, or some other generative process. As a result, they fail to make an instructive connection between their chosen characterization of abduction and the reasoning involved in EDA.

However, my major worry is not that Behrens et al. choose the wrong form of abduction to explicate the inferential nature of EDA, but that they try to understand it by appealing to abduction at all. The fundamental difference between our opposed views can be brought out by drawing, and adhering to, the important three-fold methodological distinction between data, phenomena, and explanatory theory. Briefly, data are idiosyncratic to particular investigative contexts, and they provide the evidence for phenomena, which are recurrent general features of the world that we seek to explain. In turn, phenomena are the appropriate source of evidence for the explanatory theories that we construct in order to understand empirical phenomena. In Haig (2005, 2014) I described one way of detecting phenomena by outlining a multistage model of data analysis. These stages of data analysis are concerned in turn with assessing data quality, detecting data patterns, confirming those patterns through use of computer resampling methods (a prominent feature of Tukey's conception of data analysis), and establishing the reach of the confirmed relationships in the form of inductive generalizations. Viewed in this context, EDA is an empirical, descriptive, pattern detection process. It is one component in a sequence of activities which, if undertaken successfully, can lead to the detection of new empirical phenomena.

Once claims about empirical phenomena are established, there is a natural press to understand them by constructing one or more explanatory theories. It is here, and not with the process of phenomena detection, that abduction does its work. Again, in Haig (2005, 2014) I argue how by different abductive means, one can generate explanatory theories, develop them through analogical modeling, and evaluate them in relation to their rivals in terms of inference to the best explanation. Importantly, the means I choose for showing this are, in turn, the abductive methods of exploratory factor analysis, analogical abduction, and the theory of explanatory coherence (Thagard, 1992). As methods, they provide rich abductive resources that enable researchers to produce explanatory knowledge. They well-exceed the rudimentary account of abduction provided by the above argument schema for inference to the best explanation.

Behrens et al. speak of generating hypotheses in the context of EDA. In this regard, they pose questions about things such as skewness and partialling-out. Of course, these sorts of questions can be framed as hypotheses but they are *descriptive hypotheses, not explanatory hypotheses*. They are hypotheses about data analytic matters; they are not explanations of the data patters that result from exploratory data analytic work.

*The collected works of John W. Tukey* (Vols. III and IV; Jones, 1986) provide valuable information about Tukey's wide-ranging philosophy of data analysis, including EDA. In Haig (2012), I advocate an essentially Tukeyan philosophy of data analysis. This may surprise Behrens et al., who see my philosophy as opposed to Tukey's. However, I see no tension, let alone contradiction, in subscribing to large parts of Tukey's perspective on data analysis on the one hand, and advocating a thoroughgoing abductive perspective on theory construction on the other. This is made possible by taking the compendium of exploratory data analytic methods as true to their name (they are *data analytic* methods), and abductive methods as true to their name (they are methods concerned with the construction of *explanatory* hypotheses and theories).

If researchers were to follow Behrens et al. and characterize EDA as fundamentally abductive in nature, they would risk construing descriptive hypotheses as explanatory hypotheses, when they had done no explanatory work at all. Better to put abduction to one side, and follow Tukey's philosophy of EDA.

## Conflict of interest statement

The author declares that the research was conducted in the absence of any commercial or financial relationships that could be construed as a potential conflict of interest.
